# Nanoparticle–Hydrogel Composites: Concept, Design, and Applications of These Promising, Multi‐Functional Materials

**DOI:** 10.1002/advs.201400010

**Published:** 2015-01-21

**Authors:** Praveen Thoniyot, Mein Jin Tan, Anis Abdul Karim, David James Young, Xian Jun Loh

**Affiliations:** ^1^ Institute of Materials Research and Engineering3 Research LinkSingapore117602Singapore; ^2^ Department of Materials Science and EngineeringNational University of Singapore9 Engineering Drive 1Singapore117576Singapore; ^3^ School of ScienceMonash University MalaysiaBandar Sunway47500Malaysia

**Keywords:** hydrogel‐nanoparticle composites, stimuli responsive hydrogels, polymer networks, multi‐functional materials

## Abstract

New technologies rely on the development of new materials, and these may simply be the innovative combination of known components. The structural combination of a polymer hydrogel network with a nanoparticle (metals, non‐metals, metal oxides, and polymeric moieties) holds the promise of providing superior functionality to the composite material with applications in diverse fields, including catalysis, electronics, bio‐sensing, drug delivery, nano‐medicine, and environmental remediation. This mixing may result in a synergistic property enhancement of each component: for example, the mechanical strength of the hydrogel and concomitantly decrease aggregation of the nanoparticles. These mutual benefits and the associated potential applications have seen a surge of interest in the past decade from multi‐disciplinary research groups. Recent advances in nanoparticle–hydrogel composites are herein reviewed with a focus on their synthesis, design, potential applications, and the inherent challenges accompanying these exciting materials.

## 1 Introduction

Wichterlie and Lim^1^ reported the first synthetic hydrogels with control of properties such as swelling and shrinking over several orders of magnitude. This initial discovery provided the foundation for stimuli‐responsive systems. The cross‐linked polymer network was highly sensitivity to stimuli such as solvent composition, solutes, pH, temperature, electric field, and light. This behavior of synthetic hydrogels has been reviewed elsewhere.^2^ Parallel to these developments in hydrogels and their eventual reach of the biomedical and consumer care market, several types of nanoparticle and their composites have also been being developed over decades various material research groups. Today, nanoparticles find their applications in common consumer products and appliances due to their differences in properties compared to bulk materials. This trend has generated public debate on the safety of nanoparticle technology and regulatory authorities have intervened in several countries.^3^ The challenges in the application of nanoparticles could potentially be overcome by incorporation into hydrogels resulting in decreased risks to human health and the environment. In addition, innovative combination of these two entirely different types of materials was thought to generate not only structural diversity but also a plurality of property enhancements. Such property enhancements were the main focus of research on hydrogel‐nanoparticle composite materials that resulted in improved mechanical strength and stimuli response. For example, recently reported silica nanoparticle‐hydrogel composite made of silica nanoparticles and modified poly ethylene glycol demonstrated remarkable improvements in tissue adhesive property, mechanical stiffness and bioactivity compared to hydrogel without nanoparticles.^4^ Similarly significant changes in mechanical property and thermal response were observed in poly *N*‐isopropyl amide hydrogels when gold nanoparticles immobilized in the gel.^5^ Thus the benefits of the combination of two different materials viz., nanoparticle and hydrogels lead to advanced materials with unique properties absent in the individual components. This uniqueness has catalyzed intense research activity at the interface of nanoparticle hydrogel composites looking forward to numerous applications over the past decade.

One of the earliest investigations of such materials was reported by the Willner group,^6^ in which gold nanoparticles (Au‐NPs) were immobilized in polyacrylamide (PAAm) by swelling the dehydrated gel in the presence of Au‐NP solution, resulting in uniform distribution of the Au‐NPs in the gel matrix. Consequently, various approaches for realizing these structurally unique dispersions have been reported by researchers investigating potential applications in biomedicine and optics, optics etc. Structurally similar nano‐dispersions of Au in PAAm hydrogels were obtained by in situ reduction of gold (III) chloride in the hydrogel network.^7^

**Figure 1:**
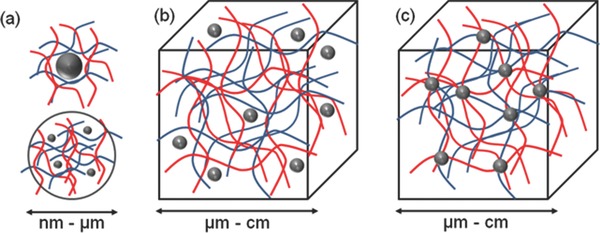
Concept for combination of nanoparticles and hydrogel to form new functional materials. Three different structural designs exist: a) micro‐ or nano‐sized hydrogel particles stabilizing inorganic or polymer nanoparticles, b) nanoparticles non‐covalently immobilized in a hydrogel matrix, and c) nanoparticles covalently immobilized in hydrogel matrix.

Three different supramolecular hydrogel‐nanoparticle designs can be proposed. They are: a) micro or nano‐gels stabilizing single/multiple nanoparticles, b) nanoparticles non‐covalently immobilized in an hydrogel matrix, and c) nanoparticles covalently immobilized in an hydrogel matrix (**Figure** **1**). There are reviews on type (a) structures covering their applications in biomedicine (particularly drug delivery), catalysis and electronics.^8^ Bulk hydrogels of type (b) and (c) have been reviewed by Schexnailder and Schmidt,^9^ but since then there have been few accounts covering only very specific applications.^10^

The current review categorizes various approaches to the design of structurally diverse nanoparticle‐hydrogel composites. It will hopefully provide a tool for selecting an appropriate method for achieving a desired nanoparticle‐hydrogel composite. The potential applications and challenges in developing these materials are presented in parallel.

## 2 Design, Synthesis, and Properties

**Figure 2:**
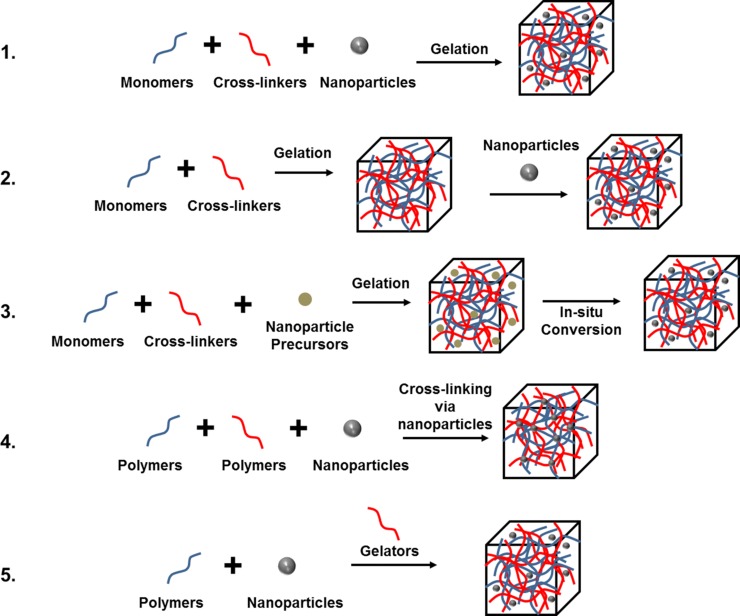
Five main approaches used to obtain hydrogel‐nanoparticle conjugates with uniform distribution: 1) hydrogel formation in a nanoparticle suspension, 2) physically embedding the nanoparticles into hydrogel matrix after gelation, 3) reactive nanoparticle formation within a preformed gel, 4) cross‐linking using nanoparticles to form hydrogels, 5) gel formation using nanoparticles, polymers, and distinct gelator molecules.

A diverse range of nanoparticle–hydrogel composites have been developed with varying types of nanoparticle embedded in a bulk hydrogel framework. Five main approaches have been used to obtain a uniform distribution: 1) hydrogel formation in a nanoparticle suspension, 2) physically embedding the nanoparticles into hydrogel matrix after gelation, 3) reactive nanoparticle formation within a preformed gel, and 4) cross‐linking using nanoparticles to form hydrogels, and 5) gel formation using nanoparticles, polymers, and distinct gelator molecules. A schematic illustration of these five approaches is given in **Figure** **2**. The approach chosen will be, in part, determined by the final application of nanoparticle‐hydrogel composite.

### 2.1 Hydrogel Formation in a Nanoparticle Suspension

**Figure 3:**
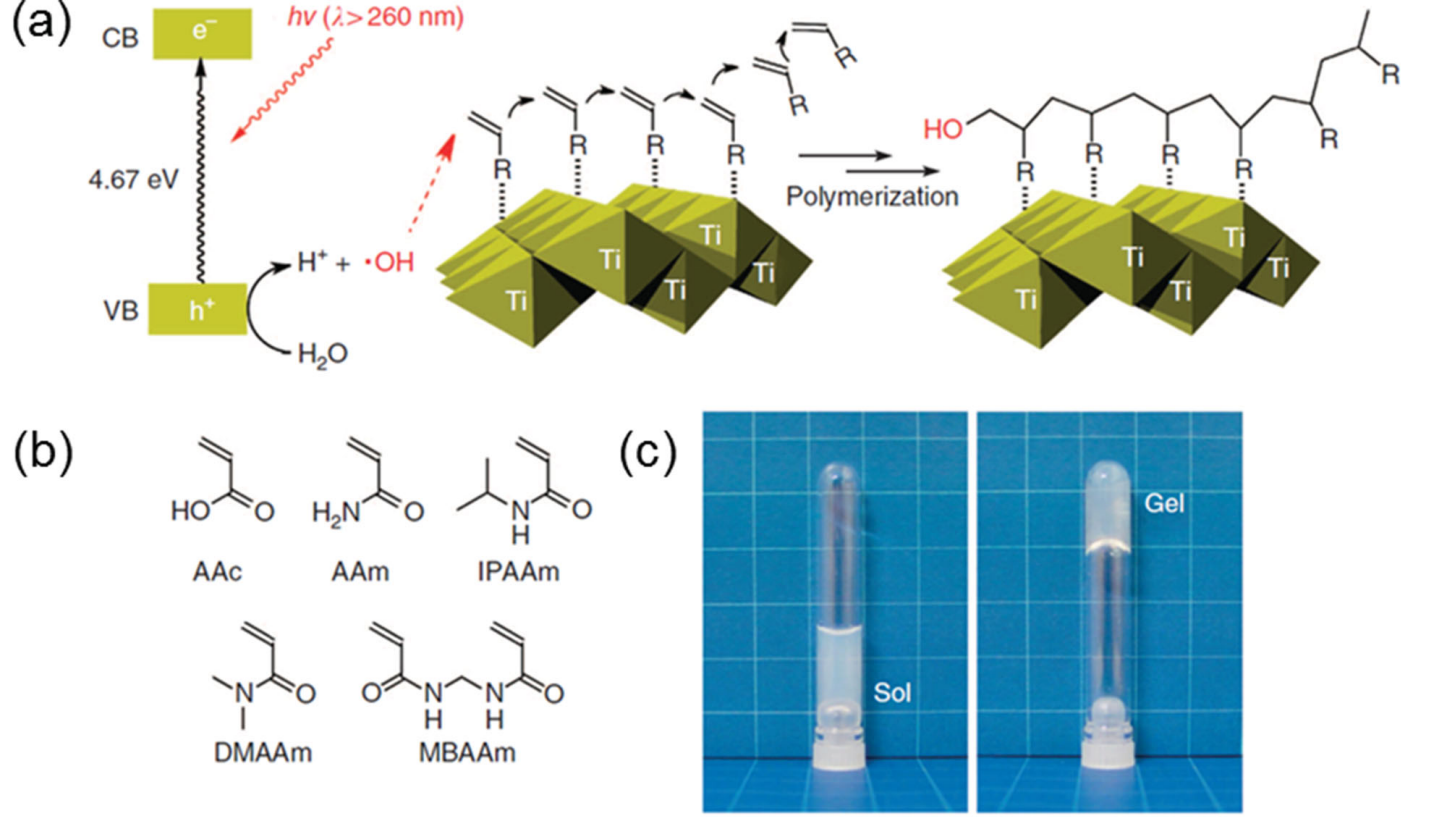
a) Schematic of titania nano sheets (Ti) acting as photo catalyst for gelation (difference in energy level of valence band and conduction band is shown). UV radiation with wavelength below 260 nm will produce hydroxyl free radicals causing gel formation reaction with vinyl monomers. b) List of vinyl monomers used for photo induced hydrogelation. c) Pictures before and after the hydrogelation using IPAAm (10.0 wt%) as monomer. The examples show hydrogel formation in a nanoparticle suspension. The nanoparticles are retained in the hydrogel by non‐covalent interactions. Reproduced with permission.^15^ Copyright 2013, Macmillan Publishers Ltd.

The simplest approach to forming a nanoparticle–hydrogel composite is the gelation of a suspension of pre‐formed nanoparticles in a hydrogel‐forming monomer solution. This approach has been utilized to form optically responsive opto‐mechanical nanoparticle‐hydrogel composites.^11^ In an example of this approach, S. R. Sershen et al.^11^ prepared gold nanoparticle (Au‐NP) hydrogel composites by adding nanoshell Au‐NPs into a solution of monomers (95/5 molar ratio of *N*‐isopropylacrylamide/acrylamide (NIPAAm/AAm) followed by addition of the gelation initiator ammonium persulfate (APS) and accelerator tetramethylethylenediamine (TMEDA). The composite was subsequently cured. Ravi et al. used this approach to incorporate three different types of nanoparticles: i) proteo‐mimetic PAAm nano‐gel, ii) bovine serum albumin, and iii) hydrophilized silica (Si), into a hydrogel matrix for intra‐ocular lens applications.^12^ Other groups have adopted this simple approach to obtain hydrogels containing Au or Si‐NPs and thereby prevent aggregation.^13^ This approach has certain drawbacks including the leaching of nanoparticles out of the hydrogel matrix if the cross link density is low.^14^ An advanced application of an hydrogel‐nanoparticle composite using a similar protocol was reported by Liu et al. for synthesizing photo‐modulable thermo‐responsive hydrogels using unilamellar titania nanosheets (TiNSs) as photocatalytic cross linkers (**Figure** **3**).^15^ In this synthesis, it was noted that the nanoparticles act as photo‐catalysts rather than cross‐linking agents and the use of bisfunctional monomer *N*,*N*'‐methylenebisacrylamide (MBAAm) was necessary for the formation of mechanically durable hydrogels.

### 2.2 Physical Incorporation of Nanoparticles into a Hydrogel Matrix after Gelation

**Figure 4:**
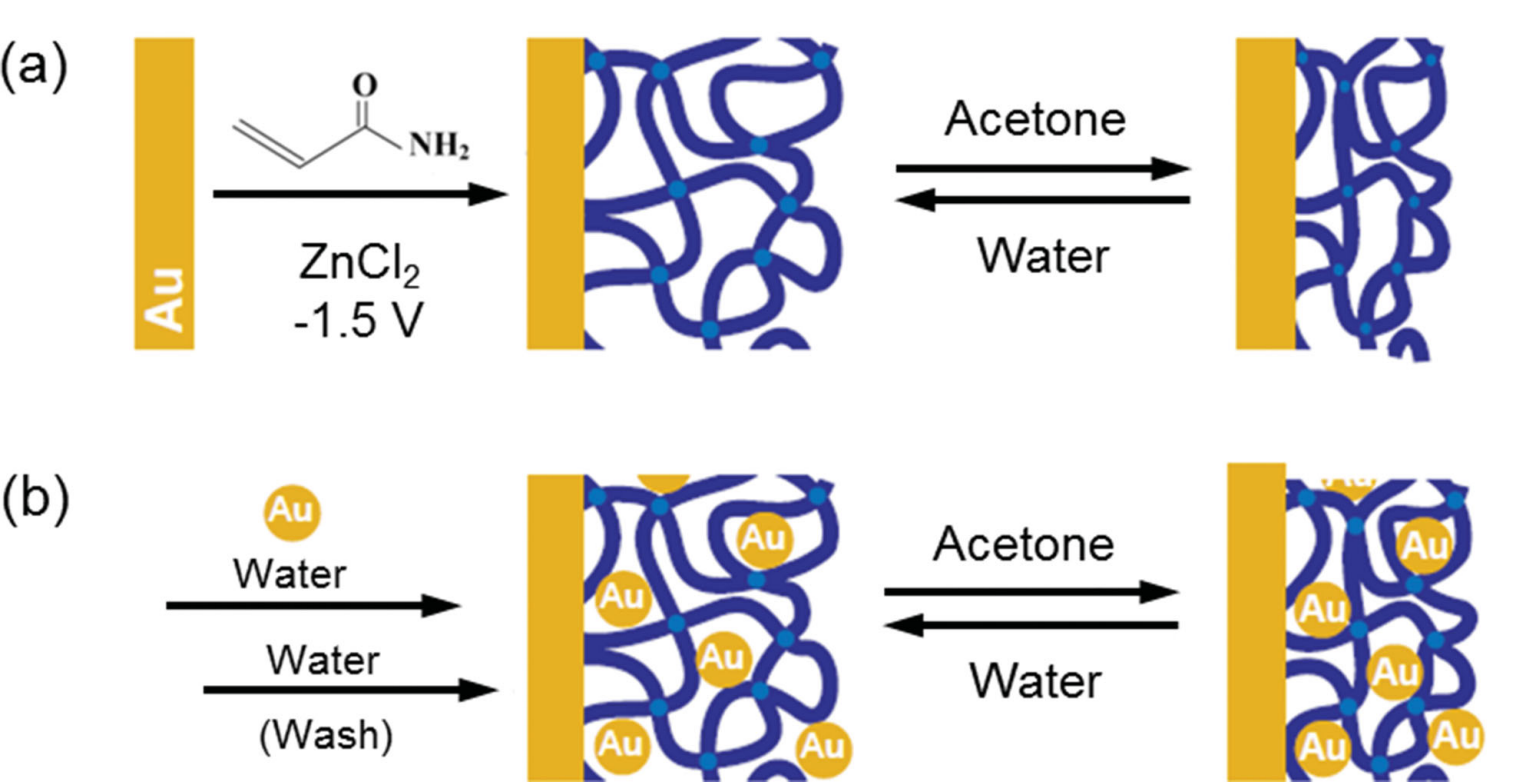
The construction of a gold‐nanoparticle/hydrogel composite at the electrode interface by switching between its swollen and shrunken states. a) Electrochemical formation of acrylamide hydrogel film at the gold electrode showing its shrinking behavior in acetone and swelling behavior in water. b) Use of alternate cycles of swelling of the hydrogel gold nanoparticle suspension in water followed by shrinking in acetone to physically entrap nanoparticles in the hydrogel. Adapted with permission.^6^

In order to study the solvent switchable electronic properties of hydrogel‐Au‐NPs, Pardo‐Yissar et al.^6^ incorporated Au‐NPs into a PAAm gel after the electro polymerization formation of the hydrogel. Electro polymerization cannot be performed in the presence of the Au‐NPs because they readily aggregate under the influence of an electric field. To counter this problem, nanoparticles were introduced into the gel matrix after the gel had already been formed via a “breathing in” mechanism. PAAm gels are highly swollen in aqueous solution, but shrink dramatically in an aprotic solvent such as acetone. Introduction of nanoparticles into the gel “breathing” consisted of three steps, which were repeated several times to obtain the desired nanoparticle density (**Figure** **4**): a) the swollen gel was placed in acetone for 2 min, causing the gel to collapse with the expulsion of water (breathing out). b) the shrunken gel was then placed in an aqueous solution of citrate‐stabilized 13 nm diameter Au‐nanoparticles (ca. 5 nM) for 2 min. This aqueous solution caused swelling of the gel in solution (breathing in), including breathing in the suspended nanoparticles. Finally, the gel was washed thoroughly with water to remove any weakly surface‐adsorbed nanoparticles. Upon the next “breathing out” cycle, the nanoparticles remain attached inside the gel, possibly due to i) physical entanglement and ii) hydrogen bonding interactions between the polymer chains and the citrate surface of the nanoparticles. These researchers confirmed the increasing presence of Au‐NPs in the gel with each “breathing” cycle by X‐ray photoelectron spectroscopy (XPS) and atomic absorption spectroscopy (AAS) measurements.^16^ Guo et al. used a similar “breathing in” approach to form highly dispersed Au‐NPs in porous anodic aluminium oxide (AAO) films by incorporating Au and platinum (Pt) NPs in PAAm hydrogels, followed by calcination.^17^ Alternatively, embedding of nanoparticles into pre‐formed colloidal micro‐gels could be achieved by repeated heating, centrifugation and re‐dispersion followed by annealing.^18^ In a typical procedure, a solution containing various ratios of micro‐gel and colloidal Au was placed into a centrifuge tube and centrifuged. Au‐NP solution was added to the micro‐gel pellet obtained and re‐dispersed throughout the micro‐gel pellet by repeated heating, agitating, and sonication. The suspension was then re‐centrifuged and the protocol repeated before a final annealing.

### 2.3 Reactive Nanoparticle Formation Aided by the Hydrogel Network

**Figure 5:**
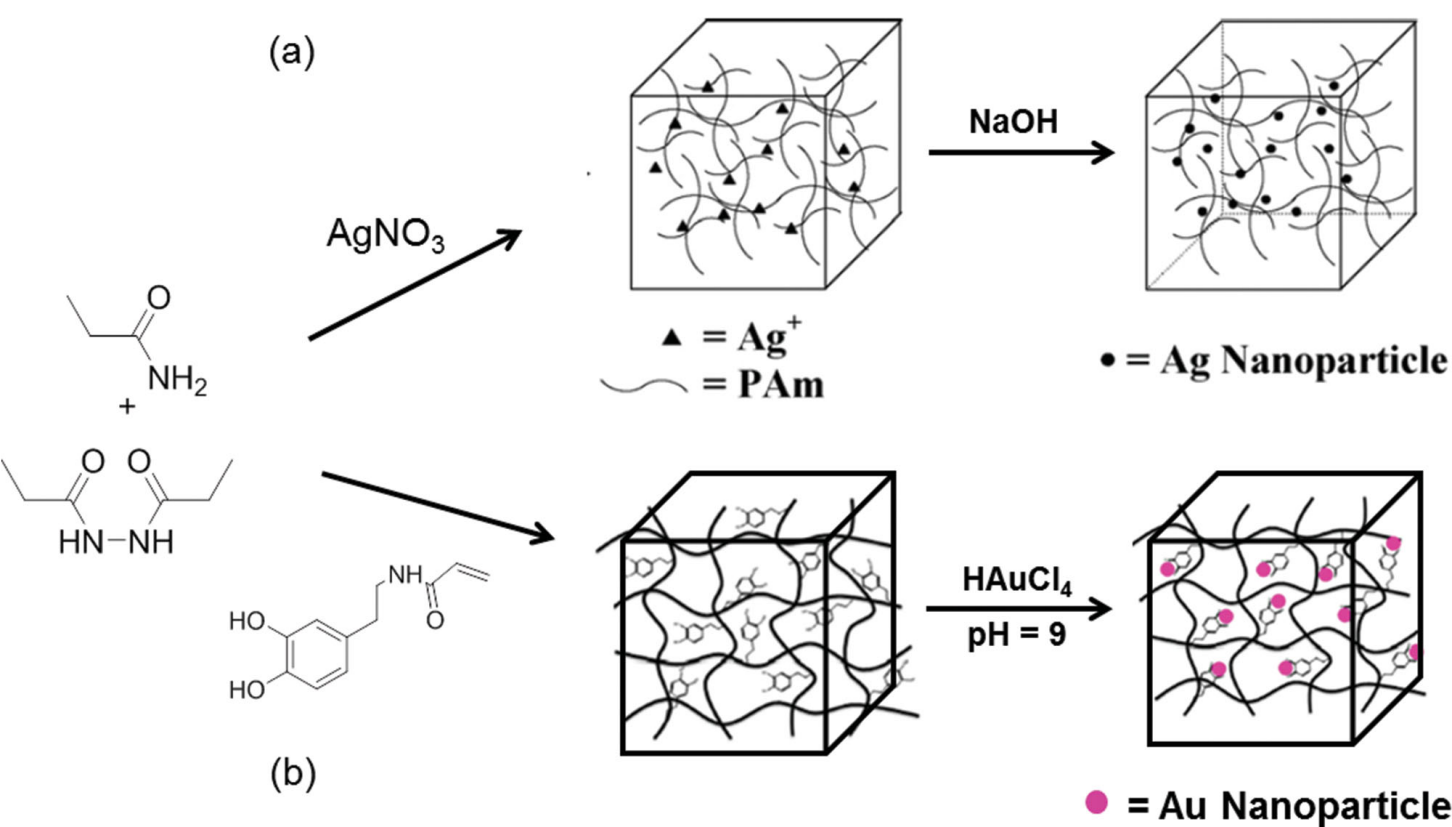
a) Preparation of Ag/PAAm hydrogel nanocomposite without the use of thiols. Reproduced with permission.^19^ Copyright 2007, Elsevier. b) Hydrogel formation and functionalization with Au NPs to obtain catalytic hydrogels by redox active catechol groups. Adapted with permission.^5^ Copyright 2014, American Chemical Society.

Langer's group developed this approach, which involves loading nanoparticle precursors into a gel, rather than preformed nanoparticles.^7^ In a typical procedure, crosslinking NIPAAm and co‐monomers containing thiol groups formed a hydrogel network containing embedded Au (III) ions. The thiol‐functionalized hydrogel matrix enabled the modulation of Au‐NP formation when a reducing agent such as sodium borohydride was added. The resulting hydrogel contained un‐aggregated nanoparticles throughout the matrix. An improved procedure of nanoparticle formation within a gel without the use of thiol or phenol containing monomers was reported by Saravanan et al. (**Figure** **5**).^19^ Free‐radical cross‐linking polymerization of acrylamide monomer in an aqueous medium containing Ag^+^ ions was conducted. The Ag^+^ ions functionalized‐PAAm hydrogel matrix was then hydrolysed to yield Ag NPs within the hydrogel network. The size of the Ag NPs in the hydrogel were estimated to be 4–7nm and well‐dispersed. The intensity of surface plasmon bands display a symmetrical configuration with no band shift and proportional increases with the increase of Ag+ ion concentration. This showed that the size of Ag nanoparticles did not vary on changing the Ag+ ion concentration and aggregation of particles are not present. These conclusions were confirmed with the electron microscopy results. This protocol has also been used for generating silver nanoparticles in interpenetrating network hydrogels.^20^ Adopting a similar method, Xiang et al. synthesized pH‐responsive Ag‐NP/poly(2‐hydroxyethyl methacrylate (HEMA)‐poly(ethylene glycol) methyl ether methacrylate (PEGMA)‐methacrylic acid (MAA)) composite hydrogels by reducing Ag^+^ ions anchored to free carboxylate groups in the matrix.^21^ In a recent study, Varaprasad et al. reduced Ag^+^ ions using green reducing agent (mint leaf extract) in a Carbopol 980 NF and acrylamide gelation mixture to produce composite hydrogels for antibacterial applications.^22^ In another recent example, Gema, M. et al. utilized redox active catechol side chain in acrylamide‐NIPAAm hydrogels to reduce gold precursor to nanoparticles to form nanoparticle‐hydrogel composites that mimic mussel adhesive protein.^5^ A schematic of this approach is presented in Figure 5(b). These hydrogels were readily formed without an external reducing agent, and great reinforcement of mechanical property was observed for the resulting composite hydrogel.

### 2.4 Cross‐Linking using Nanoparticles to Form Hydrogels

One particularly interesting example in the development of nanoparticle‐hydrogel composites involves the use of cross‐linking groups present on the nanoparticle surface. Souza et al. produced bacteriophage molecular networks by the spontaneous assembly of phage with Au‐NPs. The resulting hydrogel network preserved the cell surface receptor binding and internalization attributes of the peptide.^23^ The spontaneous arrangement of these networks could be further manipulated by incorporation of imidazole (Au–phage–imid), which induced changes in morphology, fractal structure and near‐infrared optical properties. The capacity to form multiple bonds within the gel networks (multivalency) would be a major advantage of nanoparticles as cross‐linkers, rather than the two covalent bonds of a traditional hydrogel formation reaction. This concept was well demonstrated with collagen gel formation using the 1‐ethyl‐3‐(3‐dimethylaminopropyl)carbodiimide (EDC) crosslinking reaction of mercaptopropionyl glycine protected Au‐NPs.^24^ Similarly, Zhao et al. used co‐polymerization of vinyl functionalized Au‐NPs to synthesize well‐dispersed Au‐NP‐PNIPAAm hydrogel composites with thermo‐switchable electrical properties.^25^ The transition temperature of the composite could be adjusted from 0 °C to 40 °C by changing the concentration of Au‐NPs, the degree of cross‐linking or the stoichiometry of the composite.

**Figure 6:**
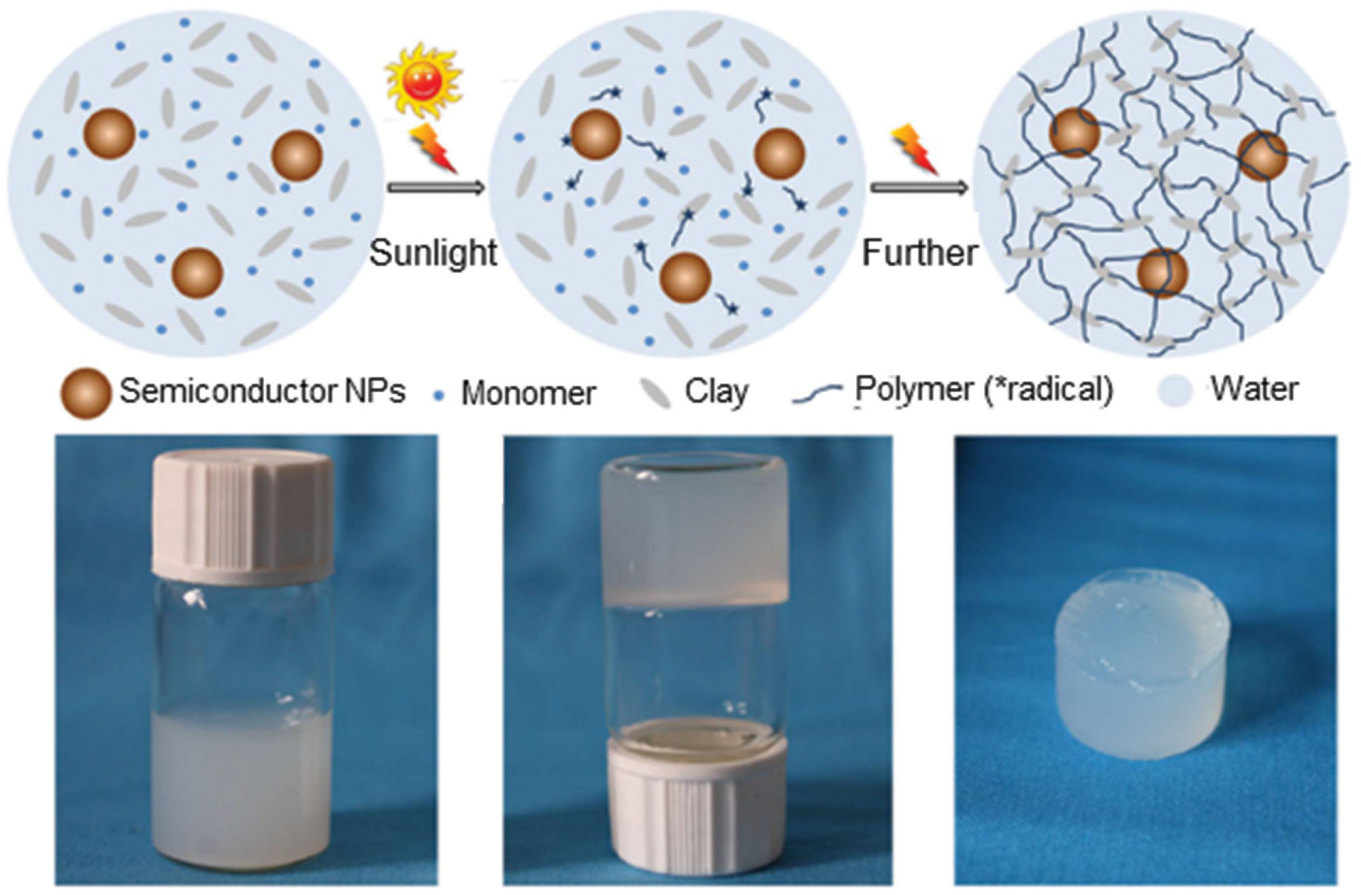
Crosslinking using nanoparticles to form nanoparticle‐hydrogel composites. Cross‐linking using clay nanostructure (Clay‐NS) to form nanoparticle‐hydrogel composites with enhanced mechanical properties. The semiconductor NPs, monomer, and Clay‐NS are homogeneously dispersed in water. Upon photo activation semiconductor nanoparticles produce free radicals initiating polymerization and crosslinking through clay‐NS. The photograph of the vials depicts optical images of the hydrogelation process. A mixture solution of ZnO nanoparticles, DMAA (N,N‐dimethylacrylamide), and Clay‐NS before gelation, hydrogelation after 1 h of irradiation and the resultant elastic ZnO nanocomposite hydrogel taken out of the vial are shown. Adapted and reproduced with permission.^15^ Copyright 2014, Macmillan Publishers Ltd.

Prestwich et al. exploited the multivalency and thiophilicity of Au‐NPs to crosslink commercially available thiolated hyaluronic acid into printable, extrudable, cytocompatible and biodegradable hydrogels.^26^ In a very recent study, Zhang et al. synthesized semiconductor nanoparticle‐based hydrogels by self‐initiated polymerization under light irradiation.^27^ The system consisted of four components: i) water, ii) water soluble semiconductor nanoparticles of zinc oxide (ZnO), titanium dioxide (TiO_2_), iron(III) oxide (Fe_2_O_3_), tin dioxide (SnO_2_), zirconium dioxide (ZrO_2_), cadmium selenide (CdSe) or cadmium telluride (CdTe), iii) *N*,*N*‐dimethylacrylamide (DMAA), and iv) clay nanosheets. The semiconductor nanoparticles functioned as inorganic initiators for the polymerization of DMAA. The authors demonstrated that CdSe and CdTe were able to form stable gels even under irradiation by visible light. The mechanism of gel formation and their visual and microscopic properties is shown in **Figure** **6**. Recently, incorporation of silica nanoparticles were shown to result in increased interfacial binding between network and nanoparticles leading to increased stiffness as well as excellent energy dissipation capability[[qv: 4b]] with orders of magnitude improvement in fracture resistance in compressive loading. The versatility of using nanoparticles as cross‐linking agent was further developed by Rose et al. for adhesion between two hydrogels.^28^ The authors demonstrated that strong, rapid adhesion between two hydrogels could be achieved at room temperature by adding a droplet of a nanoparticle solution on to the surface of the gel and bringing the other gel into contact with it. This method relied on i) the nanoparticles' ability to be adsorbed onto the polymer gels, ii) to act as a connector between polymer chains and iii) on the ability of the polymer chains to reorganize and dissipate energy under stress when adsorbed onto the nanoparticles. Silica nanoparticles (Si‐NPs), surface‐modified carbon nanotubes or cellulose nano‐crystals were suitable for this purpose. Two samples of biological tissue (e.g., cut pieces of calf's liver) could be glued together using Si‐NPs. This technology could have potential applications in surgery or tissue engineering.

### 2.5 Using Nanoparticles, Polymers and Distinct Gelator Molecules

**Figure 7:**
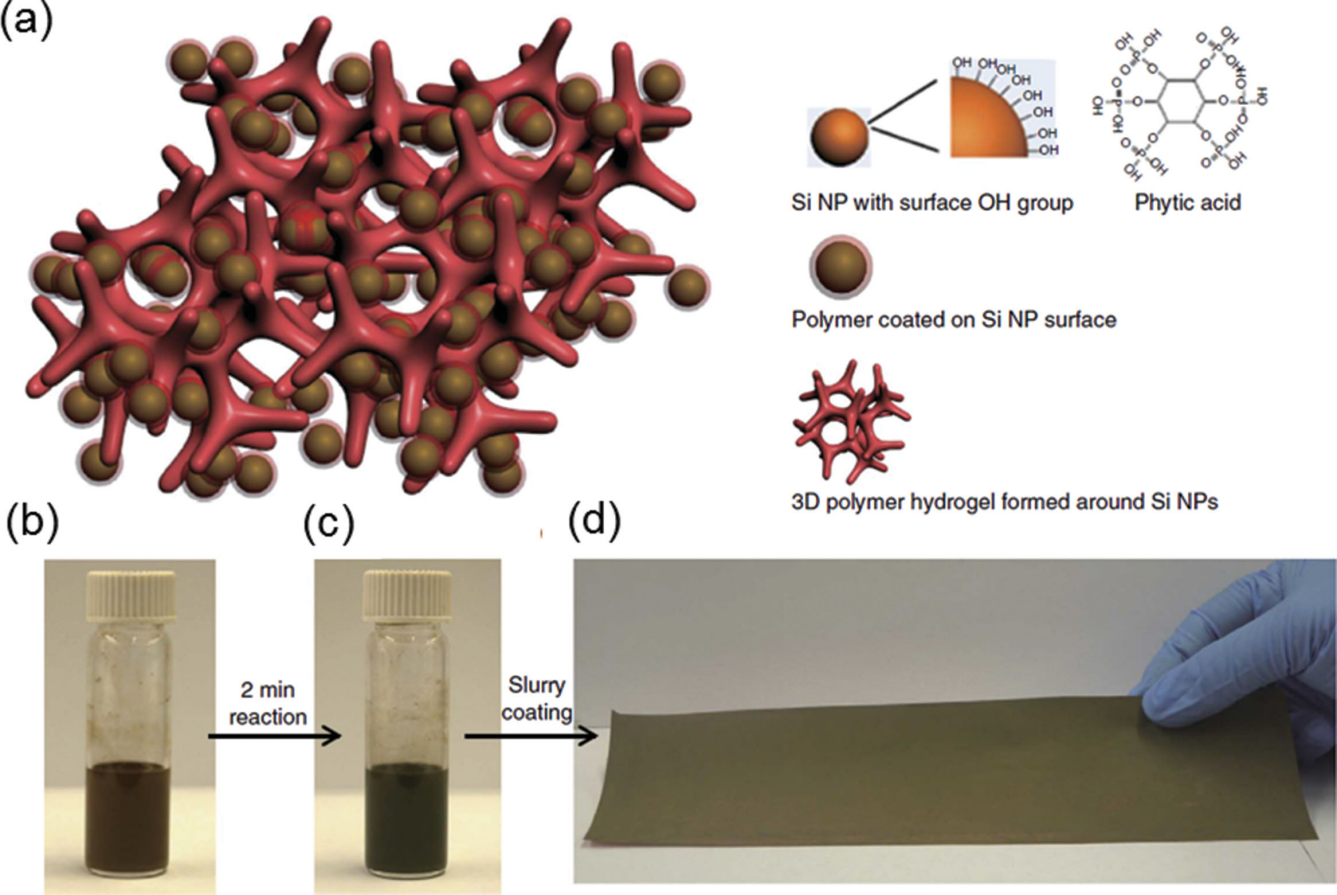
Nanoparticle‐Hydrogel composite with gelator molecules to form electrodes. a) Each (silicon‐nanoparticle) Si‐NP is encapsulated within a conductive polymer surface coating and is further connected to the highly porous hydrogel framework. b) Dispersion of Si‐NPs in the hydrogel precursor solution containing the crosslinker (phytic acid), the monomer aniline and the initiator ammonium per sulphate. c) Cross‐linked viscous gel formed after several minutes of chemical reaction, d) the hydrogel gel bladed onto a 520 cm^2^ copper foil current collector and dried to form the electrode. Reproduced with permission.^29^ Copyright 2013, Macmillan Publishers Ltd.

Wu et al. has reported incorporation of Si‐NPs into a conducting polymer hydrogel for Si‐based anodes.^29^ The hydrogel was polymerized in situ to produce a well‐connected three dimensional network structure consisting of Si‐NP coated with the conducting polymer. This hydrogel framework combined multiple positive features, including a continuous electrically conductive polyaniline network, binding with the Si surface through either hydrogen bonding with phytic acid or electrostatic interaction with the positively charged polymer, and porous space for volume expansion. Formation of this hydrogel was achieved with a scalable solution phase synthesis by mixing Si NPs with phytic acid and aniline in water followed by the addition of an oxidizer (for example, ammonium persulphate). The aniline oxidised rapidly and polymerized to form cross‐linked polyaniline and the mixture formed a dark green viscous gel due to the presence of the phytic acid gelator. The viscous gel was then bladed onto a copper foil current collector and dried to form a uniform film over a large area for electrochemical applications (**Figure** **7**).

## 3 Types of NP‐Hydrogel Composites and Their Applications

The innovative combination of nanoparticles and hydrogels create synergistic, unique and potentially useful properties that are not found in the individual components. Properties imparted to the composites depend on the type of nanoparticles incorporated, which in turn is determined by the proposed application of the designed composite. Different types of nanoparticle‐hydrogel composites and their associated properties and applications are described below.

### 3.1 Metal NP‐Hydrogel Composites

#### 3.1.1 Silver NP‐Hydrogel Composites

**Figure 8:**
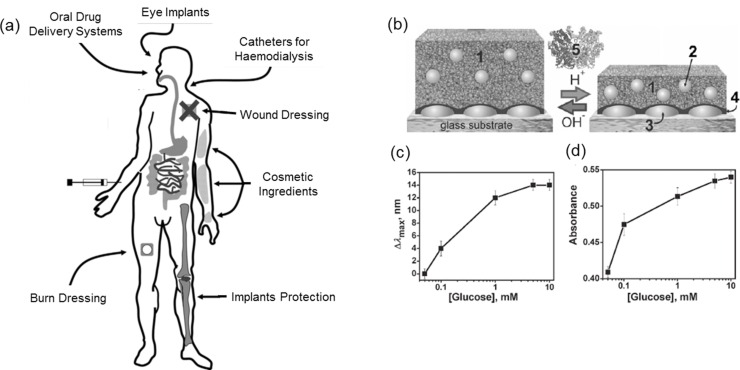
a) Overview of potential medical applications for Ag NP‐hydrogel composites. Reproduced with permission.^37^ Copyright 2013, Elsevier. b) Ag NPs incorporated into pH‐responsive hydrogel based glucose oxidase activity sensor. c) Shifts in silver nanoparticle absortion maxima (Δλ_max_) as functions of glucose concentration for the plasmonic sensing device. d) Spectrophotometric glucose sensing. Change in absorption at 470 nm. Adapted with permission.^48^

Silver (Ag) NPs are known for their antimicrobial properties and have been widely used in dental fillings and, more recently in wound and burn dressings to prevent infections.^30^ Ag‐NPs bind non‐specifically to bacterial membranes and other components, inducing structural changes that increase membrane permeability and mitochondrial dysfunction.^31^ Controlled release of Ag‐NPs is necessary to sustain antimicrobial efficacy. As such, design of Ag NP‐hydrogel composites was expected to provide functional coatings for various applications (**Figure** **8**a).^32^ Furthermore, properties such as mechanical toughness, swelling ratio, stimuli responsiveness and bio‐compatibility/degradability would need to be investigated and optimized in such composites for effective application. Ag‐NPs have been incorporated into PAAm,^33^ polyacrylic acid (PAA),^34^ NIPAAm,^35^ methyl methacrylate^36^ and polyvinyl alcohol (PVA) based hydrogels.^37^ These Ag NP‐hydrogel composites show promise as functional anti‐microbial coatings. Semi‐interpenetrating network (IPN) hydrogels offer an excellent alternative for applications requiring higher mechanical toughness. These materials also acted as templates for the synthesis of small (2–5nm) and uniformly distributed Ag‐NPs.^20^ Efforts in recent years have shifted to utilizing naturally occurring materials such as chitosan,^38^ carbohydrate polymers such as gum acacia and dextran^39^ and gelatin^40^ to produce bio‐compatible/degradable composite materials that have potential applications as implantable dressings. The controlled‐release of Ag‐NPs provides consistent protection for a period of time, without the need to remove the dressings. Tokarev et al. demonstrated that Ag‐NPs enhance the efficacy of surface plasmon resonance (SPR)‐based sensors.^41^ Ag‐NPs incorporated into pH‐responsive hydrogels with the enzyme glucose oxidase function as glucose concentration sensors (Figure 8b). The swelling‐shrinking transition of Ag‐NP filled hydrogels alter the inter‐particle distance and so affect the optical response of SPR‐based sensors, leading to sensitive glucose detection.^42^ Additionally, Ag‐NPs have been incorporated into hydrogels to form electrically conducting hydrogels. The relationship between initial precursor Ag^+^ ions concentration and swelling ratio of the hydrogel have a direct impact on conductivity. A higher concentration of Ag^+^ ions resulted in better conductivity, but reduced swelling ratios, and vice versa.^19^ High conductivity without affecting the swelling ratio could potentially be achieved with Ag nanowires (1D nano‐structures) rather than Ag NPs (0D nano‐structures).^43^

#### 3.1.2 Gold NP‐Hydrogel Composites

**Figure 9:**
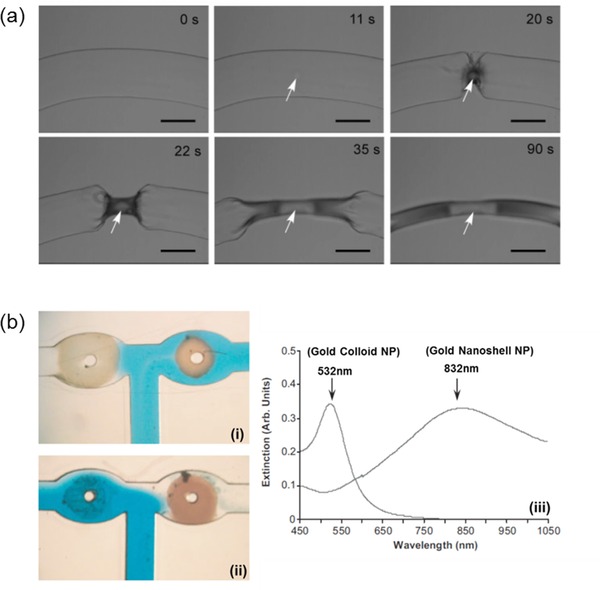
a) Photo‐thermal effect of laser irradiated Au‐nano rods, causing localized collapse of hydrogel. White arrow indicates position of Au‐nano rods and laser irradiation. Reproduced with permission.^57^ Copyright 2007, American Chemical Society. b) T‐junction of a microfluidic device with Au‐colloid hydrogel (right valve) and Au nano shell hydrogel (left valve) with 100 μM wide channels i) When the device is illuminated with 532nm green light the left valve opened ii) when the device is illuminated with 832nm IR light the right valve opened. iii) The absorption spectra of gold nano particles and gold nano shells. Adapted and reproduced with permission.^50^

Stimuli‐responsive and switchable conductive Au‐NP‐hydrogel composites have been demonstrated by several groups.^25^, ^44^ External stimuli such as temperature or pH cause a change in conductivity of the hydrogel due to the change in inter‐particle distance. Even though Au‐NP‐hydrogel composites have shown efficacy in SPR based sensors^45^ and anti‐bacterial applications,^46^ the high cost of gold has so far prevented wide‐scale adoption of Au‐NPs for such applications. Irradiation of light at the Au plasmonic peak induces localized heating within a temperature‐responsive gel matrix.^47^ This phenomenon can be used for remote‐controlled drug delivery. If the temperature rises above the lower critical solution temperature (LCST) of the gel matrix, the gel structure collapses resulting in an on‐demand burst release of drugs as opposed to a diffusion‐controlled release. Shiotani and co‐workers have demonstrated this concept using a system (**Figure** **9**a) of Au nanorods‐NIPAAm composite hydrogels and rhodamine‐based materials.^48^ They reported a fast and reversible shrinking and re‐swelling of this composite hydrogel. The fast response was attributed to heat generation within the gel matrix, rather than external to the matrix. Cytotoxicity studies of these composite gels on cultured cells are underway, with clinical trials expected in the near future.^48^ Different sized or structured Au‐NPs immobilized in a thermo‐responsive hydrogel matrix can also function as remote‐controlled microfluidic valves. The principle behind this application relies on the variation of thermal response with nanoparticle size.^49^ The use of different excitation wavelengths renders opening of different valves when the correct amount of energy is delivered by matching the plasmonic resonant peaks of the NPs. On irradiation with 532nm light, Au‐colloids (3–10 nm diameter) with plasmonic peak at 532nm collapse the hydrogel network, opening the valve. Valves containing Au‐nanoshell (diameter 120nm, shell thickness 10nm) hydrogel composites remain unaffected. Opening of these valves containing Au‐nanoshells could be achieved upon illumination at 832nm (Figure 9b).^50^

#### 3.1.3 Other Metal NP‐Hydrogel Composites

Although the majority of metal NP‐hydrogel composite studies involve Ag and Au‐NPs, several other metallic NPs also show promise in various fields including catalysis, magnetic components and environmental nanotechnology. Platinum (Pt) metal is well‐known as a hydrogenation catalyst. Pt‐NPs in a bola‐amphiphile hydrogel was found to be an efficient catalyst for the hydrogenation of p‐nitroaniline.^51^ This procedure can be used for templated, in situ synthesis of nanoparticles leading to uniform distribution and high loadings. Magnetic NPs of cobalt (Co) or nickel (Ni) have also been incorporated into hydrogels to form soft, magnetic field driven actuators for muscle‐like applications.^52^ Ni and Ni‐nickel oxide core‐shell NPs incorporated into PVA hydrogels were responsive to magnetic fields and used for separating and concentrating of chemical species from a mixture.^53^ Copper (Cu) has been investigated as a cost effective alternative to Ag and Au‐NP anti‐microbial agents. Cometa and co‐workers have demonstrated an effective Cu‐NP‐poly(ethylene glycol diacrylate) hydrogel anti‐microbial coating, where the combined effect of the high surface/volume ratio of Cu‐NPs and charged quaternary ammonium salts led to potent bactericidal activity.^54^ In situ synthesis of bi‐metallic NPs provides interesting nanocomposite hybrid materials. Fe‐Co bimetallic NPs, for example, were synthesized in a PAAm hydrogel network to form magnetic hydrogels for waste removal applications.^55^ Poly(2‐acrylamido‐2‐methyl‐1‐propansulfonic) acid has been used as a template for the synthesis of Co:Ni bimetallic NPs, which can catalyse the hydrolysis of sodium borohydride (NaBH_4_) for hydrogen production and are also magnetically responsive, to be sequestered when not required.^56^ Zhang et al. recently used a poly(ethylene oxide propylphosphonamidate) (PEOPPA) hydrogel bearing multi‐amine groups to carry out in situ reduction with concomitant nanoparticle formation. Uniform immobilization of noble metal nanoparticles (Au, Ag, Pd, Pt and Ru) were obtained in the absence of any other reducing agents and stabilizers.^57^ These nanoparticle‐infused hydrogels were used for the reduction of nitro aromatics in the presence of NaBH_4_. This system showed excellent recyclability with retention of catalytic activity because aggregation induced deactivation was prevented by the hydrogel network.

### 3.2 Metal Oxide NP‐Hydrogel Composites

**Figure 10:**
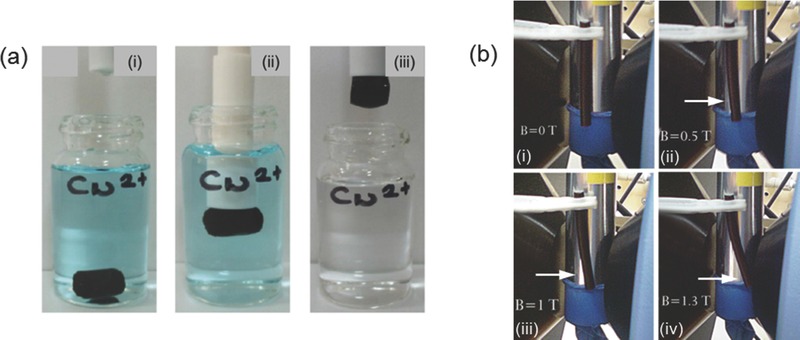
a) Removal of Cu2+ ions in aqueous medium, i) Ferrogel in solution of Cu2+ ions, ii) Magnetic rod attracts ferrogel, iii) Removal of ferrogel is accompanied by the removal of Cu^2+^ ions as well. Reproduced with permission.^65^ Copyright 2010, Elsevier. b) Bending of ferrogel under varying magnetic field. i) No bending under no field. (ii–iv) Increasing bending observed as magnetic field is increased from 0.5T to 1.3T. Magnetic field strength is indicated in the pictures. Reproduced with permission.^66^

Metal oxide NPs hydrogel composites have been developed for their ferromagnetic and semi‐conducting properties. Iron oxide (FexOy) is a commonly synthesized ferromagnetic material that has recently been incorporated into hydrogels to form ferrogels.^58^ Ferrogels made from poly(acrylamide‐co‐maleic acid),^59^ pNIPAAm,^60^ 4‐vinylpyridine,^61^ chitosan blends with PAA^62^ and methacrylate co‐polymers^63^ loaded with magnetite NPs are effective absorbents for toxic ions such as lead (Pb^2+^), chromium (Cr^2+^) and arsenic (As^5+^). The embedded pollutants can then be collected magnetically.^64^ Similarly, Ozay et al. (**Figure** **10**a) used a poly(2‐acrylamido‐2‐methyl‐1‐propansulfonic acid‐co‐vinylimidazole) hydrogel loaded with Fe(II)oxide magnetite NPs to absorb and remove Cu^2+^ ions from solution.^65^ Ferrogels can also be magnetically driven actuators to mimic muscle movement. Caykara and co‐workers prepared Fe_3_O_4_ NPs immobilized in poly(N‐tert‐butylacrylamide‐co‐acrylamide) hydrogels by in situ oxidation of iron precursor in an alkaline medium.^66^ These ferrogels displayed actuation under magnetic field (Figure 10b), suggesting that further research could lead to devices exhibiting human‐like actuation. Magnetite NPs embedded in thermo‐responsive hydrogels can be employed for drug delivery and microfluidic valve control in a similar fashion to that described above for light activated Ag and Au NP composites.^38^ In this case, alternating magnetic fields (AMFs) result in a localized temperature change around the NP, stimulating the hydrogel matrix.^67^ Titanium oxide and ZnO NPs, have been developed for their UV protective and photocatalytic properties. These materials have potential applications in skin care products and self‐cleaning surfaces.^68^

### 3.3 Non‐Metal NP‐Hydrogel Composites

**Figure 11:**
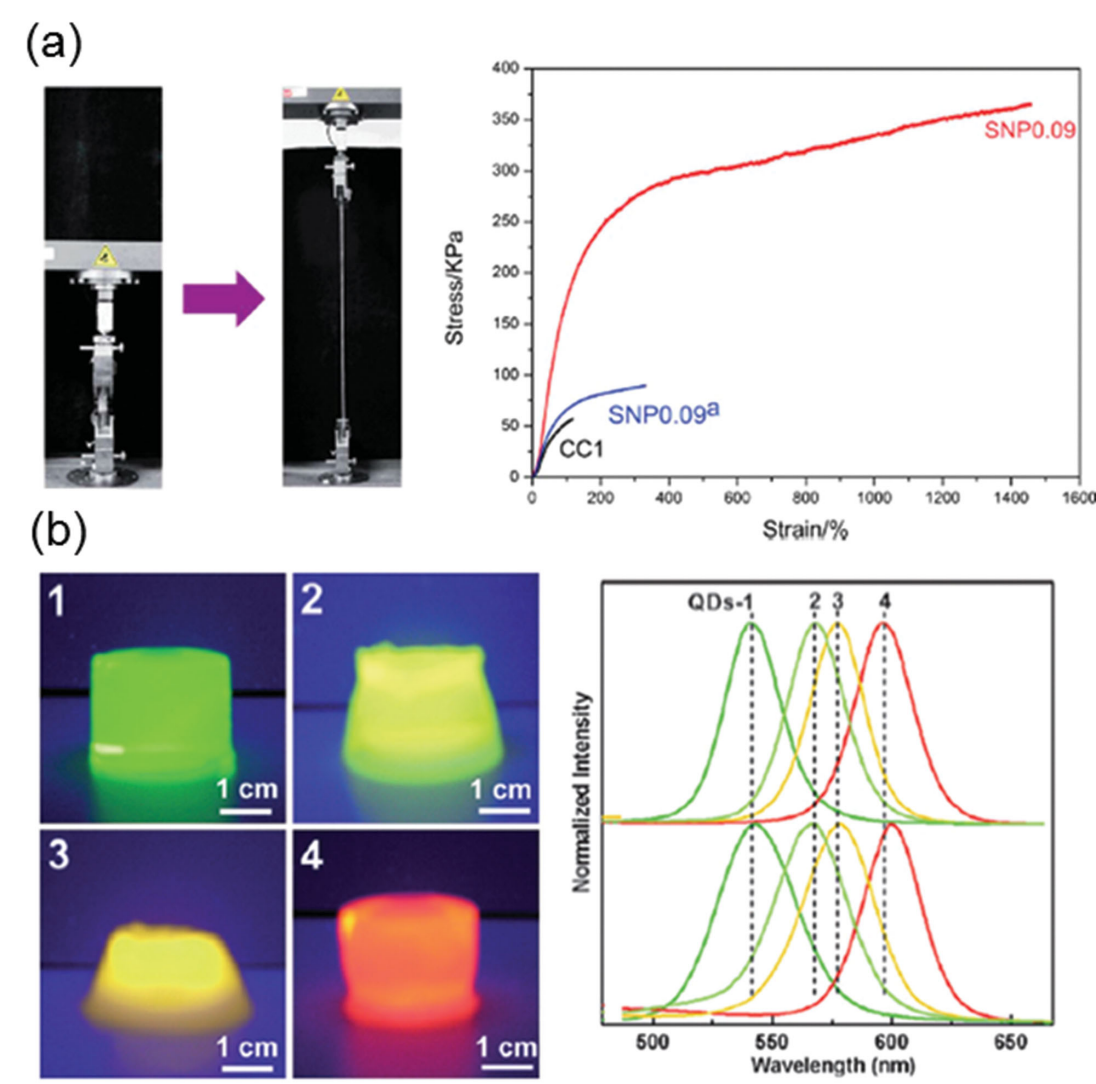
a) Left: Physical demonstration of extreme elasticity of Si‐NPs hydrogels by stretching. Right: Stress‐strain plots showing excellent toughness by Si‐NP hydrogels. SNP 0.09 indicate gels made with 0.09mg/mL surface treated silica nanoparticle, SNP0.009^a^ indicate gels made with same amount of silica without surface treatment. CC1 is the hydrogel without silica nanoparticles. Reproduced with permission.^69^Copyright 2013, Royal Society of Chemistry. b) Demonstration of cellulose networks in the hydrogels protecting the CdSe/ZnS structure and preserving the quantum dots characteristics. Appearances of the Quantum dot (QD) ‐cellulose hydrogels under a 302 nm UV lamp (left), and Photoluminescence spectra of CdSe/ZnS (core/shell) QDs with average diameter 2.8 nm (green), 3.0 nm (yellowish‐green), 3.2 nm (yellow) and 3.6 nm (red) respectively in buffer solution. QD‐cellulose hydrogel emission peaks are matching with (right, bottom) with emission peaks of the free quantum dots in buffer. Adapted and reproduced with permission.^77^ Copyright 2009, Royal Society of Chemistry.

Non‐metal based NPs such as carbon‐based materials (graphene oxide, nanodots, nanotubes), quantum dots (CdTe) and Si have been used to create composite materials with unique properties and functions. Si‐NPs have been traditionally used as a support for catalysts or functional materials. Yang et al. incorporated Si‐NPs as cross‐linking centers in PAA based hydrogels that resulted in excellent mechanical strengthening (**Figure** **11**a).^69^ Si‐NP‐hydrogel composites have also been shown to have higher drug release efficiency when loaded with a drug such as doxorubicin.^70^ Alvarez and co‐workers developed an antibiotic loaded (gentamicin) Si‐NP‐collagen composite hydrogel that exhibited prolonged antibacterial activity and increased mechanical strength.^71^ Similarly, Lee and co‐workers used Si‐NPs‐collagen hydrogel composites for delivering nerve growth factors for neural tissue engineering.^72^ Annaka and co‐workers developed a hydrophobically modified polyethylene glycol containing an hydrophilized Si‐NPs hydrogel composite for injectable intraocular applications.^73^ In a similar approach Liu et. al, designed an injectable tissue adhesive using dopamine‐modified four‐armed poly(ethylene glycol and with a synthetic nanosilicate, Laponite.[[qv: 4a]] A subcutaneous implantation study of these materials in rats demonstrated improvement in mechanical and adhesive properties with enhanced cellular infiltration and minimum inflammatory response. Carbon nanotubes,^74^ graphene oxide^75^ and even melanin,^76^ when placed in hydrogels act as NIR light absorbing materials useful for photo‐thermal drug delivery. These materials present synthetic alternatives to Au or Ag‐NP based remote‐controlled photo‐thermal systems. Semiconductor quantum dots CdSe/ZnS and CdSe/CdS have been incorporated into hydrogels to produce fluorescent hydrogels bio‐markers. Chang and co‐workers prepared highly fluorescent, robust cellulose‐QD hydrogels (Figure 11b)^77^ while Salcher and co‐workers made CdSe/CdS QDs/PNIPAAm hydrogel beads for cell labelling.^78^

### 3.4 Polymeric NP‐Hydrogel Composites

**Figure 12:**
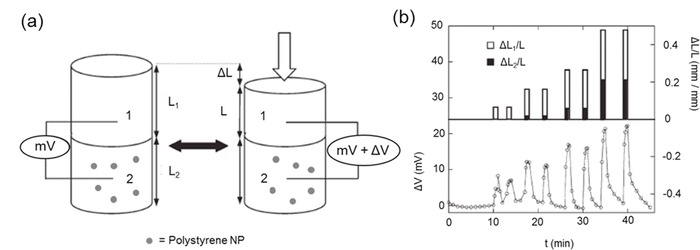
a) Mechanical compression of bilayer gels with and without poly styrene nanoparticles (PSNPs) Gel 1: without PS NP (top) and Gel 2: with 26% PS NP (bottom part of the cylindrical gel) are attached together and compression is given from top (arrow). b) Time profile of deformation ΔL_1_/L and ΔL_2_/L (top) and electric potential ΔV generated (bottom) upon deformation. Reproduced with permission.^89^ Copyright 2007, Royal Society of Chemistry.

Polymeric NPs composed of micelles,^79^ nano‐gels,^80^ core‐shell particles,^81^ dendrimers,^82^ hyper‐branched polymers,^83^ and liposomes^84^ have been developed for a variety of applications. The inclusion of these particles in a hydrogel imparts multi‐functionality because the polymer particles themselves possess multiple functional groups. Many designs have biomedical applications including drug delivery and bio‐sensing. Zhong et al. enhanced the biological stability of collagen by the physical incorporation of poly amidoamine dendritic NPs.^85^ Such physical incorporation resulted in enhanced mechanical properties due to the numerous interactions within the hydrogel network which, in turn, increased human conjunctival fibroblast proliferation. Zhang et al. overcame the limitation of poor drug loading and lack of control over the lower generation dendritic NPs by using generation 5, hyper‐branched polyamine ester nanoparticle in an hydrogel network.^86^ These hydrogels allowed week‐long, controlled release of active ingredients which was not possible with hydrogels that did not possess NPs. The use of polymeric NPs for reinforcement of hydrogels is not restricted to biomedical applications. Polystyrene (PS), a material commonly used for packaging and storage, could be used as filler in hydrogels to impart mechanical strength.^87^ Bait et al. developed an acrylamide and hydroxyethyl methacrylate hydrogel with PS‐NP fillers for superior elasticity in dermatological patches.^88^ Thevenot et al. developed hybrid materials with differing elastic moduli by using alternating layers of PAAm hydrogel composites with and without PS‐NP fillers. Physical deformation of this bi‐layer gel produced an electrical potential, useful in developing soft tactile‐sensing devices (**Figure** **12**).^89^ Polypyrrole (PPy) is a semi‐conducting conjugated polymer used in organic‐based optoelectronic devices. Luo et al. developed an agarose/alginate double network hydrogel incorporating PPy NPs for use as an infrared responsive releasing system, much like those employing reduced graphene oxide NPs.^38^

## 4 Conclusions and Outlook

We have reviewed nanoparticle‐hydrogel composites as a state‐of‐the‐art, versatile class of materials suitable for a wide range of applications. Synthetic methods and strategies and the unique synergistic properties of the composite that is absent in individual components, together with their applications, have been summarized. Nanoparticle‐hydrogel composites exhibit multi‐functional and stimuli responsive properties, making them ideal for “smart” materials, including i) antimicrobial gels/barriers/matrices; ii) photo thermally active hydrogels; iii) soft material catalysts; iv) environmental absorbents; v) drug delivery vehicles; vi) optical detection sensors and vii) soft actuators. Potential applications include i) safe, clinically implantable nanoparticle‐hydrogel composite systems for bio‐sensing and therapy; ii) environmental remediation systems for catalytic oxidation of toxins/removal of pollutants; iii) recyclable catalytic nanoparticle hydrogel composites for chemical synthesis and iv) composite hydrogel patches for cosmetic applications.

We expect that the classification of synthetic approaches and applications of nanoparticle‐hydrogel composites presented in this review provide a better understanding of the system and enable the reader to design innovative combinations for new applications. Control of the covalent and supra molecular interactions by synthetic design and prediction of the resultant properties in the nanoparticle‐hydrogel composite are major areas to be developed in this emerging field. Such predictions upon experimental validation will form the foundation of design for the next generation nanoparticle‐hydrogel composites with optimal properties for a desired application. In the coming years, such design of nanoparticle‐hydrogel composites will not only result in advanced applications, but will also steer the fundamental understanding of material interactions, aiding computational prediction of properties of new composites from existing components.
